# A functional profile of gene expression in ARPE-19 cells

**DOI:** 10.1186/1471-2415-5-25

**Published:** 2005-11-01

**Authors:** Rajesh K Sharma, William E Orr, Allyson D Schmitt, Dianna A Johnson

**Affiliations:** 1 Department of Ophthalmology and Hamilton Eye Institute, University of Tennessee Health Science Center, 930 Madison Ave, Memphis, TN 38163, USA

## Abstract

**Background:**

Retinal pigment epithelium cells play an important role in the pathogenesis of age related macular degeneration. Their morphological, molecular and functional phenotype changes in response to various stresses. Functional profiling of genes can provide useful information about the physiological state of cells and how this state changes in response to disease or treatment. In this study, we have constructed a functional profile of the genes expressed by the ARPE-19 cell line of retinal pigment epithelium.

**Methods:**

Using Affymetrix MAS 5.0 microarray analysis, genes expressed by ARPE-19 cells were identified. Using GeneChip^® ^annotations, these genes were classified according to their known functions to generate a functional gene expression profile.

**Results:**

We have determined that of approximately 19,044 unique gene sequences represented on the HG-U133A GeneChip^® ^, 6,438 were expressed in ARPE-19 cells irrespective of the substrate on which they were grown (plastic, fibronectin, collagen, or Matrigel). Rather than focus our subsequent analysis on the identity or level of expression of each individual gene in this large data set, we examined the *number *of genes expressed within 130 functional categories. These categories were selected from a library of HG-U133A GeneChip^® ^annotations linked to the Affymetrix MAS 5.0 data sets. Using this functional classification scheme, we were able to categorize about 70% of the expressed genes and condense the original data set of over 6,000 data points into a format with 130 data points. The resulting ARPE-19 Functional Gene Expression Profile is displayed as a percentage of ARPE-19-expressed genes.

**Conclusion:**

The Profile can readily be compared with equivalent microarray data from other appropriate samples in order to highlight cell-specific attributes or treatment-induced changes in gene expression. The usefulness of these analyses is based on the assumption that the numbers of genes expressed within a functional category provide an indicator of the overall level of activity within that particular functional pathway.

## Background

The retinal pigment epithelium (RPE) is a monolayer of hexagonal cells separating the neural retina from the underlying choroidal vascular bed. RPE cells are essential for development, survival, and physiological activity of photoreceptor cells [1,2]. RPE cells provide the molecular machinery for recycling the inactive form of the photoisomerized visual pigment back to the active isomer for subsequent formation of rhodopsin [3]. RPE phagocytizes spent photoreceptor outer segments; provides nutrients to, and removes metabolic waste from, the photoreceptors; contributes to retinal adhesion and maintenance of the blood-retinal barrier; and absorbs light and dissipates heat energy derived from incident light [4,5]. Recent evidence shows that RPE cells also participate in the immunologic functions in the retina. RPE cells can express major histocompatibility complex (MHC) class I and II antigens and the intercellular adhesion molecule-1 (ICAM-1). These cells process and present the antigen to helper T cells [6-9]. RPE responds to proinflammatory cytokines and secretes IL-6, IL-8, and monocyte chemotactic protein [10-14]. Through these mechanisms RPE cells play a key role in inflammatory, infectious, and degenerative diseases of the retina. Impairment of RPE functions have been implicated in a number of hereditary retinal degenerations [15-18], and more importantly in the pathogenesis of age-related macular degeneration (AMD), one of the most prevalent causes of visual impairment in elderly [19]. Given the importance of RPE cells in the normal physiology and disease of retina, RPE has become the subject of intense investigation especially those elucidating the role of RPE cells in the molecular mechanisms of AMD. Transplantation of normal as well as genetically modified RPE cells is being envisaged as a possible treatment of retinal degenerations [20,21]. Given the pivotal role of RPE in retinal development, physiology and diseases it is important to investigate the gene expression profile of these cells, which will than lay the foundation for further molecular characterization of RPE cells in both normal and diseased states.

DNA microarray technology provides a view of the expression profiles of a cell sample that encompasses virtually the entire genome. Microarray technology has a number of distinct applications including DNA sequencing, mutation analysis, gene discovery, and gene expression analysis [22-26]. Microarray technology allows a rapid quantitative measurement of gene expression within a tissue sample, as defined by messenger RNA (mRNA) abundance. The opportunity to quantitate gene expression on a genome-wide scale has added a new dimension to our understanding of many biologic and disease processes. However, analysis of large data sets derived from microarray analysis can be problematic. It is an overwhelming task to consider the expression levels of each of the twenty or so thousand known genes *individually*. An alternative strategy is to group individual genes into functional categories in order to generate what has been termed a "functional gene profile". Different types of analyses can then be applied to gene profiles. For example, functional categories of genes displaying the highest levels of expression can be identified and thus provide a means for focusing on groups of functionally related genes that may be highly expressed by a specific cell type or physiological state. "Cluster analysis" is another more complex type of gene profile analysis in which significant changes in gene expression due to some experimental variable are mapped with respect to functional categories.

We propose a novel approach to gene profile analysis based on simply the total *number *of genes within a functional category that are expressed above some pre-determined level. In order to meet the goal of generating a data set that will be comprehensive but not too large to be readily useful, we have generated a functionally classified list of genes whose expression level met the specific criteria established by Affymetrix analysis instead of considering the absolute level of expression. Below we describe 130 functional categories that account for 68% of the genes represented on the Affymetrix microarray chip, and 70% of the genes expressed by the ARPE-19 cell line. Key words for the functional categories were chosen from the HG-U133A GeneChip^® ^Library, allowing us to use Affymetrix annotation terms (i.e., key words) to sort genes into categories. We used this classification scheme to calculate the number of functionally related genes expressed within a category and to produce a ARPE-19 Gene Expression Profile. Since data for each category consists of a single number, the entire database for RPE gene expression can be represented in a Profile with 130 data points. We suggest that comparing the ARPE-19 Profile with the profile of genes represented on the Affymetrix HG-U133A GeneChip^® ^may provide a measure of the cell-specific pattern of gene expression unique for RPE cells. A RPE-specific expression profile data base would have a number of potential uses, such as selecting specific genes and functionally related groups of genes for further analysis with microarray and validation by RT-PCR. Data already available in the literature demonstrates that many of the genes included in our expression profile are known to be present in ARPE-19 cells as validated by quantitative RT-PCR (for example, see Chowers I 2004 IOVS [27]). We further suggest that functional categories with large numbers of expressed genes may reflect high relative importance of those specific functions to RPE. Another unique aspect of our approach was that it excluded from analysis any genes whose expression was dependent on the substrate upon which the ARPE-19 cells were grown. There is no uniformly accepted substrate utilized researchers in this field, yet expression of certain classes of genes is known to be substrate dependent. (See Discussion.) We grew cells on four commonly used substrates (fibronectin, Matrigel, collagen, and uncoated plastic culture dishes) and included in our analysis only those genes that were uniformly expressed by cells on all four substrates. Our intent was to focus on "substrate-independent" genes that would be likely to be expressed under most experimental conditions. The advantages and disadvantages related to this overall analysis strategy are reviewed in the Discussion section.

## Methods

### RPE culture

ARPE-19 cells were used in the experiments. These are diploid non-transformed human RPE cells that display many properties typical of differentiated RPE in vivo [28]. ARPE-19 cells were obtained from a commercial source (ATCC, Manassas, VA). The cells were plated on 75-cm^2 ^flasks at a density of 10,000 cells/cm^2 ^and maintained in culture until the plates became >95% confluent. Cultures were fed three times a week with Dulbecco's modified Eagle's medium-nutrient mixture F-12 (DMEM-F-12; GIBCO, Invitrogen Corporation, Grand Island, NY) supplemented with 10% fetal bovine serum, 100 U/mL penicillin, and 100 μg/mL streptomycin. Cultures were passaged by dissociation in 0.05% (wt/vol) trypsin. For the microarray experiments, cells belonging to passage 4 were used 24 hrs. after removing serum from the medium in order to further synchronize the metabolic and physiological state of cells.

### Substrate-dependent genes

Our overall goal in these experiments was to generate a functional catalogue of "core" ARPE-19 genes. To achieve that goal, we sought to avoid genes that might be highly sensitive to exact culturing conditions, such as the choice of substrate. While substrate-sensitive genes are likely to be very important to the cell, as a group they may confound our results. Since there is no uniformly accepted substrate for APRE-19 culture, we reasoned that a catalogue of genes that were expressed regardless of substrate would provide the best standard. Cells were grown on four commonly used substrates: fibronectin, Matrigel, collagen, and uncoated plastic. Each sample was run on a separate chip and analyzed as described below. Any genes not expressed on all four substrates were identified as "substrate-specific genes" and were removed from further analysis. Data from the remaining substrate-independent genes were considered as n = 4 (In other words, since we only included genes uniformly expression in all four samples, there is no variation in the gene expression profile among the n = 4). Variability in expression levels of substrate-independent genes among these four chips was relatively low (see Results) suggesting that overall variability due to technical factors was low.

### Microarray methods

The RNA isolation procedures for the Affymetrix analysis were conducted using TRIzol Reagent (GIBCO, Carlsbad, CA) according to the manufacturer's instructions. Initially, the quality of total RNA was assessed by electrophoresis through a 1% agarose gel, then the Agilent Bioanalyzer System (Agilent Technologies, Palo Alto, CA) immediately prior to cRNA synthesis. The procedures for the Affymetrix gene chips, beginning with first strand cDNA synthesis, were conducted by Genome Explorations (Memphis, Tennessee). The Human Genome U133A GeneChip^® ^contains 22,283 probe sets together with expressed sequence tag (EST) sequences. The RNA (isolated using TRIzol) was run over of a G50 spin column. First and second strand cDNA were synthesized from 15 μg of total RNA using the SuperScript Double-Stranded cDNA Synthesis Kit (GIBCO, Carlsbad, CA) and oligo-dT24-T7 (5'-GGC CAG TGA ATT GTA ATA CGA CTC ACT ATA GGG AGG CGG-3') primer according to the manufacturer's instructions. cRNA was synthesized and labeled with biotinylated UTP and CTP by in vitro transcription using the T7 promoter coupled double stranded cDNA as template and the T7 RNA Transcript Labeling Kit (ENZO Diagnostics Inc. Farmingdale NY). The fragmented cRNA was hybridized to the olygonucleotide array, washed, stained with phycoerythrein conjugated streptavidin (Molecular Probes, Eugene OR), and scanned. Intensities were determined using a laser confocal scanner (Hewlett-Packard; Palo Alto, CA).

The scanned images were analyzed using Microarray Suite Version 5.0 (MAS 5.0, Affymetrix, Inc., Santa Clara, CA). The MAS 5.0 statistical algorithms calculate signal intensity, probe set detection, probe set (gene expression) change, and signal log ratio. The signal intensity for each gene was calculated as the average intensity difference, represented by [S(PM – MM)/(number of probe pairs)], where PM and MM denote perfect-match and mismatch probes.

The analysis applied a decision matrix based on the hybridization behavior of all 11 probe pairs per probe set. These matrices are used to determine if the gene is expressed above a threshold level (i.e., called Present by Absolute Call decision matrix). Investigators can change the specificity and sensitivity criteria for the present call by changing the alpha-1 value in the MAS 5.0 software. We used a value of 0.05 as a standard and 0.07 or 0.18 for less restrictive present calls.

Many genes on the chip are represented by more than one probe set. Since we were interested in the number of expressed genes, not the number of probe sets, a method was devised for selecting a single probe set to represent each gene. First, we performed a search for multiple probe sets using the designated Unigene ID or title for each gene. Once the redundant probe sets were identified, we determined the coefficient of variation of the expression levels of each probe set for the n of 4 chips. The probe set with the lowest coefficient of variation was chosen. Data from replicate probe sets for the same gene were omitted from further analysis. This choice of method was somewhat arbitrary, but it had the advantage of being based strictly on a statistical criterion and it should not introduce any bias in the data since all probe sets, all genes, and all chips were treated the same. The coefficient of variation data was not used for any subsequent steps in the analysis. The resulting database, free of redundant probes for any given gene, was used for determining the number of genes expressed within functional categories.

Functional categories were established for classification of the majority of genes represented on the HG-U133A GeneChip^® ^, including those expressed by ARPE-19 cells. (See Results for additional details.) HG-U133A GeneChip^® ^annotations were downloaded from Affymetrix. Using FileMaker Pro 5.5 (FileMaker, Inc., Santa Clara, CA), the following annotations were used to create a HG-U133A GeneChip^® ^Library: Probe Set ID, Title, Unigene ID, Sequence Derived from, Sequence Description, Archival Reference Group, Gene Symbol, GO Biological Process, GO Molecular Function, Proteome Biochemical Function, Proteome Cellular Role, Interpro ID and Classification, Ortholog-Homolog, and Pathways. Relational databases were created by linking the HG-U133A GeneChip^® ^Library to the four data sets provided by the Affymetrix MAS 5.0 analysis. Search terms (i.e. keywords) were used to extract the probe sets for each functional category. The resulting classification scheme consisted of 130 functional categories that could be grouped under 15 major subheadings. Many of the genes could be classified in more than one category. Genes were included in all the categories in which they were classifiable. Additional literature searches were used to clarify ambiguities. In order to determine whether the relative number of genes expressed by ARPE-19 cells within a particular functional category was significantly different from that predicted based on numbers represented on the chip, we performed a z-test

## Results

### Numbers of genes expressed

The Affymetrix HG-U133A GeneChip^® ^contains 22,283 probe sets representing 19,044 distinct genes, of which 6,438 genes were called as present in all four samples of ARPE-19 cells with an alpha-1 value of 0.05. When present calls were made with a less restrictive sensitivity and specificity criterion (alpha-1 value was changed to 0.18), 8,671 genes were called present.

### Establishment of functional categories

**Figure 1:**
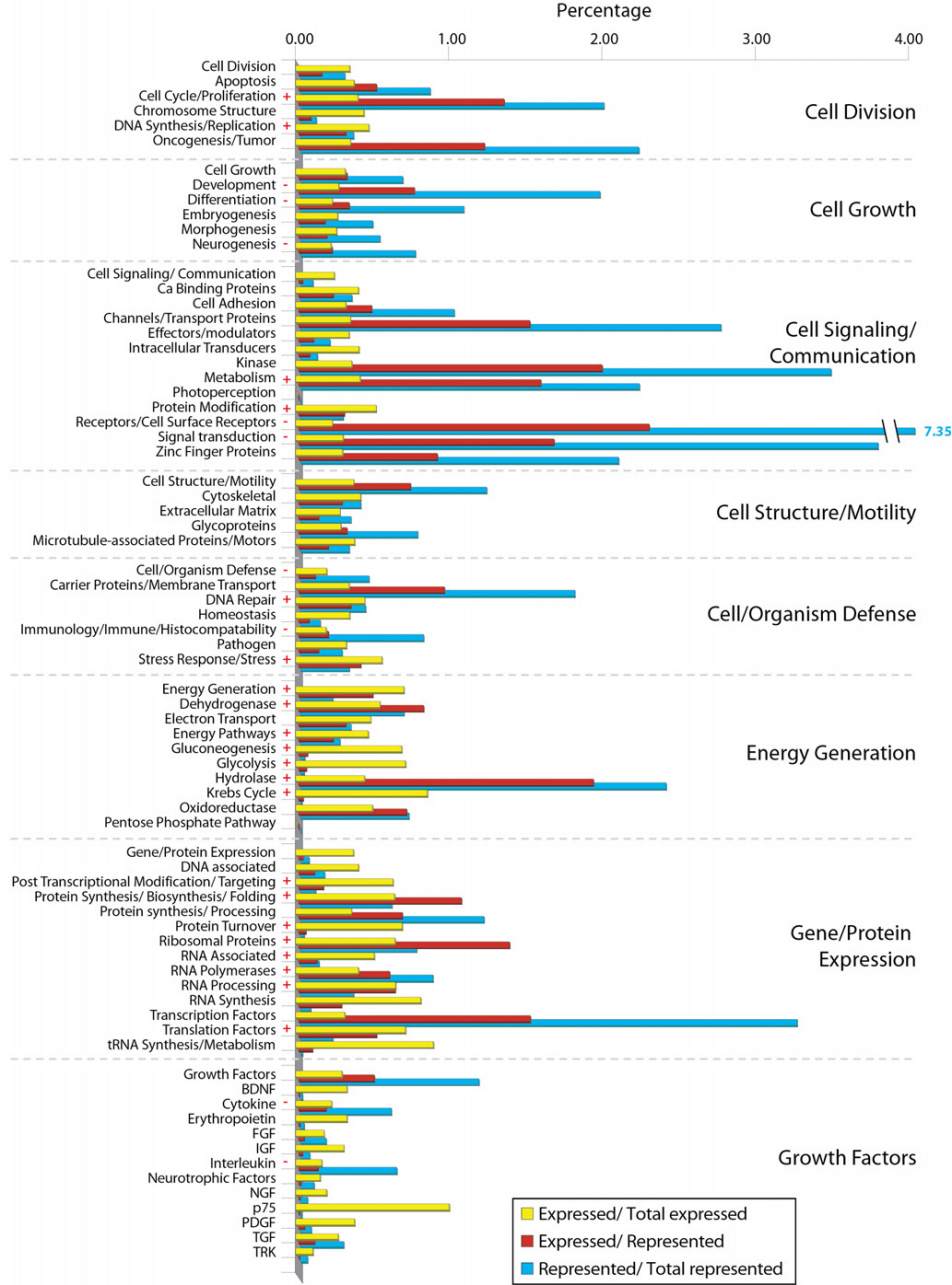
**Functional categories of genes represented on the HG-U133A GeneChip^174 ^and expressed by ARPE-19 cells**. A total 130 functional categories are displayed on the abscissa. These are arranged alphabetically in groups under 15 large functional subheadings indicated on the right and separated by dashed lines. The numbers of genes represented and expressed were converted into percentages indicated on the ordinate. The formulae used for calculating percentages were different for each color-coded bar. Yellow bar = total number of genes expressed by ARPE-19 within the functional category/total number of genes expressed in all categories. The heights of the yellow bars may reflect the relative importance of a given function in ARPE-19 cells. Red bar = total number of genes expressed within the functional category/total number of genes represented on the HG-U133A GeneChip^174 ^in all categories. Blue bar = total number of genes represented within the functional category/total number of genes represented in all categories. Comparisons of the heights of the red versus blue bars in each category show considerable variation from the overall average of approximately 33% expressed. Based on a z-test analysis, categories that have statistically higher-than-predicted percentages expressed are noted by "+;" those with statistically lower-than-predicted percentages expressed are noted by "-." These differences indicate that the functional profile of genes expressed are not a simple reflection of the functional profile of genes represented on the chip. Abbreviations: Ca – calcium; BDNF – brain derived neurotrophic factor ; IGF – insulin growth factor; TRH – thyroid releasing hormone; GABA – gamma amino butyric acid; NADPH – nicotinamide adenine dinucleotide phosphate (reduced form); 5HT – 5 hydroxytryptamine; AMPA – alpha amino-3-hydroxy-5-methylisoxazole-4-proprionic acid; NMDA – n-methyl-d-aspartate.

**Figure 2:**
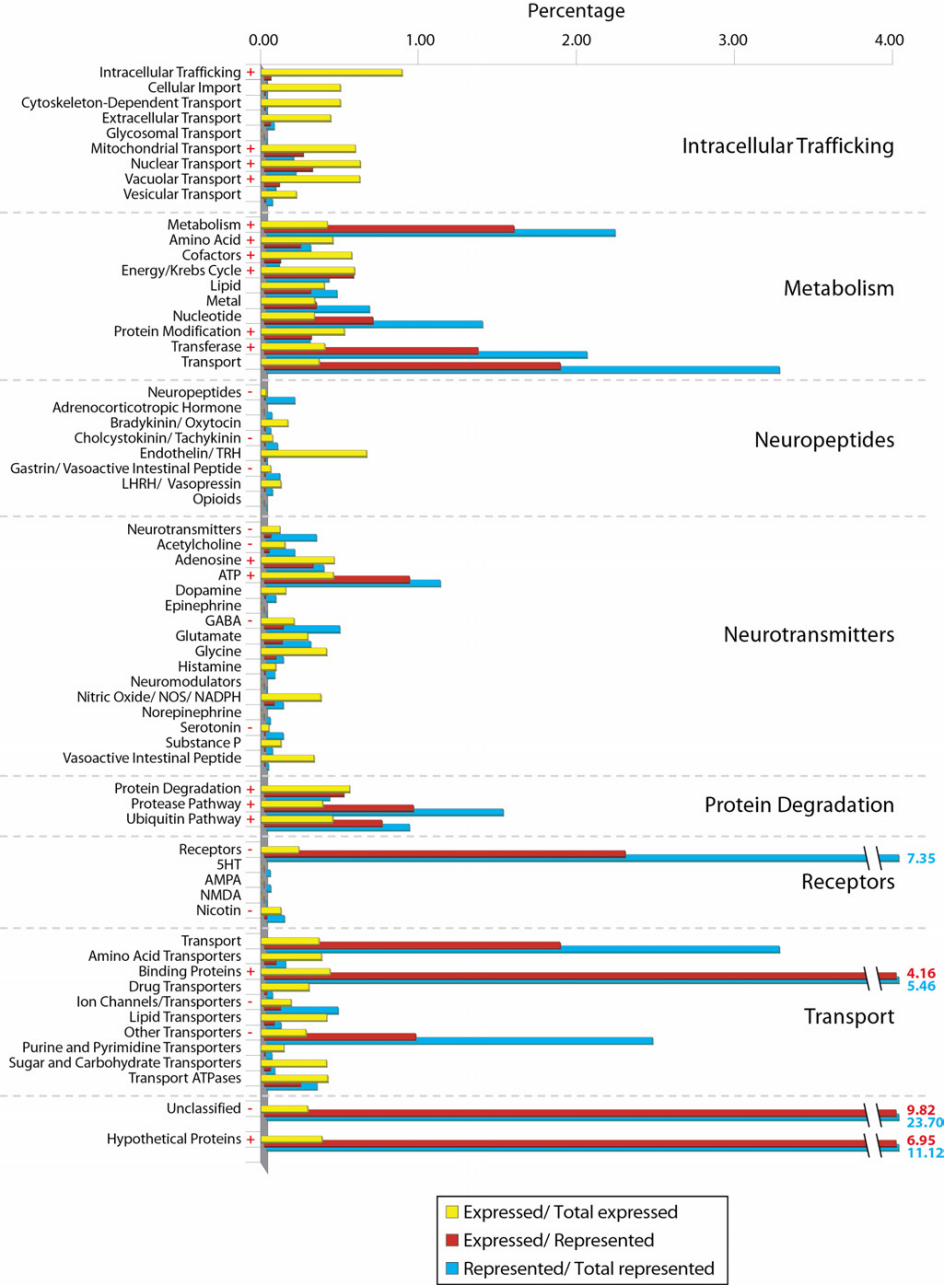
**Functional categories of genes represented on the HG-U133A GeneChip^174 ^and expressed by ARPE-19 cells**. A total 130 functional categories are displayed on the abscissa. These are arranged alphabetically in groups under 15 large functional subheadings indicated on the right and separated by dashed lines. The numbers of genes represented and expressed were converted into percentages indicated on the ordinate. The formulae used for calculating percentages were different for each color-coded bar. Yellow bar = total number of genes expressed by ARPE-19 within the functional category/total number of genes expressed in all categories. The heights of the yellow bars may reflect the relative importance of a given function in ARPE-19 cells. Red bar = total number of genes expressed within the functional category/total number of genes represented on the HG-U133A GeneChip^174 ^in all categories. Blue bar = total number of genes represented within the functional category/total number of genes represented in all categories. Comparisons of the heights of the red versus blue bars in each category show considerable variation from the overall average of approximately 33% expressed. Based on a z-test analysis, categories that have statistically higher-than-predicted percentages expressed are noted by "+;" those with statistically lower-than-predicted percentages expressed are noted by "-." These differences indicate that the functional profile of genes expressed are not a simple reflection of the functional profile of genes represented on the chip. Abbreviations: Ca – calcium; BDNF – brain derived neurotrophic factor ; IGF – insulin growth factor; TRH – thyroid releasing hormone; GABA – gamma amino butyric acid; NADPH – nicotinamide adenine dinucleotide phosphate (reduced form); 5HT – 5 hydroxytryptamine; AMPA – alpha amino-3-hydroxy-5-methylisoxazole-4-proprionic acid; NMDA – n-methyl-d-aspartate.

In order to establish a functional profile of genes, each of the 19,044 distinct genes represented on the HG-U133A GeneChip^® ^was assigned a functional classification using Affymetrix software search programs. By a process of trial and error, these individual functional classifications were organized into a limited number of function-related categories. Our goal was to establish a classification scheme that would include as many genes as possible within a "reasonable" number of functional categories. By this process, we were able to select 130 functional categories that accounted for 66.48% of the genes printed on the HG-U133A GeneChip^® ^and 70.94% of the genes expressed by ARPE-19 cells. These categories are listed on the abscissa of Figure 1 and 2, arranged under 15 major functional subheadings. Most of the genes could be classified in more than one category. Genes were counted in each functional category they were associated with, which resulted in multiple listings of most genes. Roughly 33% of the genes printed on the HG-U133A GeneChip^® ^and 29% of the genes expressed by ARPE-19 cells could not be classified in functional categories because the probes represented genes with unknown products or unknown functions. In addition, certain functional categories represented by very small numbers of genes were also placed under the category of unclassified for practical reasons.

### A functional profile of gene expression

Rather than display the data as absolute numbers of genes in each functional category, we chose to represent each number as a percentage. Results are shown in Figure 1 and 2, using percentages calculated from the formulae listed below.

*The blue bars *= the number of genes in a given category that are represented on the HG-U133A GeneChip^®^/the total number of genes represented on the HG-U133A GeneChip^® ^.

*The red bars *= the number of genes in a given category that are expressed by ARPE-19/the total number of genes represented on the HG-U133A GeneChip^® ^.

Results show that the functional profile of the genes expressed by ARPE-19 was significantly different from the functional profile of genes represented on the HG-U133A GeneChip^® ^. Overall, ARPE-19 expressed 33.8% of the represented genes. However, the percentage of genes expressed in each functional category varied considerably from that norm. Based on a z-test analysis, a total of 60 of the 130 functional categories contained significantly more or significantly less than the predicted number of expressed genes (See Fig. 1 and 2).

An additional indication of the uniqueness of the ARPE-19 functional gene profile can be seen in a comparison of functional categories containing the largest percentages of both represented and expressed genes. The ten categories containing the largest numbers of genes are shown in Table 1. These ten categories represent the same four basic functional groups in both lists: Receptors, Signal Transduction, Metabolism, and Transport. In spite of this overall similarity, these two lists differ in several important aspects. 1) Genes related to Metabolism and to Signal Transduction-associated Metabolism were among the most numerously expressed by ARPE-19, whereas these genes were not among the most numerously represented on the chip. The reverse was true for genes related to Transcription Factors. 2) Of particular interest were several cases in which ARPE-19 expressed approximately 50% or more of the genes represented on the HG-U133A GeneChip^® ^. (See Fig. 1 and 2.) This occurred in functional categories that were large (i.e., Binding Protein, Hydrolase, and Metabolism), as well as those that were small (i.e., Ribosomal Proteins and Protein Synthesis). Based on these data, we conclude that the ARPE-19 Functional Gene Expression Profile does reflect characteristics of the ARPE-19 cell type and that it is not simply a reflection of the functional groups of probe sets represented on the HG-U133A GeneChip^® ^.

We completed an additional calculation in which the Functional Gene Expression Profile was expressed as a different type of percentage also shown in Fig. 1 and 2, using the formula below:

Yellow bars *= the number of genes within a given functional category that are expressed by ARPE-19/Total number of genes expressed*.

This calculation is comparable to that used in constructing a standard "pie chart," which has been routinely used by other investigators to display the function of genes expressed in a given cell type. Inclusion of 130 functional categories used in our analysis adds considerably more detail than could be contained on a normal pie chart format and thus cannot be presented as such. However, the bar graph presentation does allow an expanded overview of virtually all known genes within a reasonably simple format. Results show that several functional categories of genes account for a large portion of the total number of genes expressed.

In the three largest functional categories (Binding Protein, Cell Surface Receptors, and Receptors), the number of genes expressed accounted for approximately 32% of the total number of genes expressed. It must be kept in mind that our classification scheme includes most genes in more than one category and that there is likely to be considerable overlap among these three categories. Thus, the actual number of distinct genes in this functional grouping could be as low as 10%. Even so, this represents a major functional class of genes expressed by ARPE-19.

In each of eight large categories, the number of genes expressed accounted for approximately 5% of the total number expressed. These latter categories represent genes associated with cell signaling, transport, gene/protein expression, and energy metabolism.

### Effect of specificity/sensitivity parameters and substrate on gene classification

In order to determine the degree to which specificity and sensitivity parameters influence the analysis and functional categorization of the expressed genes, we altered the alpha-1 value in the Microarray Suit 5.0 so that the present calls were less specific but more sensitive, i.e., less restrictive. When the alpha-1 value was changed from 0.05 to 0.075 only 570 additional genes were called present, suggesting that our data were not overly influenced by the sensitivity parameters originally selected. To increase the number of present calls by approximately 30% required setting the alpha-1 value as high as 0.18. With this alpha-1 value, 8671 genes were called present in all four samples.

To determine the degree to which our results were influenced by variations in genes expressed by samples from the four different culture substrates used, we compared the number of genes expressed in all four samples with the number of genes expressed in at least one of the samples. Results show that 6438 genes were called present in all samples and that 9749 genes were called present in at least one sample. This suggests that substrate may have significant effects on gene expression.

## Discussion

Mapping of chromosomal positions and genomic organization of human genes has elucidated the chemical background of the genome [29], linking specific genes to various human diseases. However, to understand the pathophysiological mechanisms, it is prudent to resort to functional genomics approaches [30,31]. Identifying the genes expressed in a particular tissue and profiling their function, as we have done in this study for a widely used human retinal pigment epithelium cell line, lays the foundations for such understanding.

An additional objective was to focus primarily on the genes likely to be consistently expressed even under varying culture conditions, specifically when different substrates were used. By including only substrate-independent genes, the Profile may be more widely applicable to labs using different culture conditions and substrates. There is no standard substrate that is uniformly accepted by ARPE-19 researchers; several are in common usage (plastic, collagen, Matrigel, and fibronectin). Our aim was to include genes that are expressed by the ARPE-19 cells irrespective of the substrate. We cultured four samples for microarray analysis, each on one of these four substrates and then eliminated from our analysis any genes that were not uniformly expressed by all four samples. Theoretically then, our analysis should be independent of specific substrate effects. It should be kept in mind, however, that substrate-specific effects may play an important role in RPE cells. In vivo, RPE cells grow in a penta-lamellar structure called Bruch's membrane that is thought to play an important role in the health and disease of RPE cells [1]. Changes in the Bruch's membrane have been implicated in the pathogenesis of age-related macular degeneration where RPE also plays an important role. It has been shown that attachment of RPE cells to the basement membrane is essential for its survival. All these facts imply that the nature of basement membrane affects the gene expression in RPE cells. These genes may not be included in our database of 6,438 genes called present in all four samples. For comparison, we calculated that 9,749 genes were expressed by at least one of the four samples. This suggests that up to 3,311 genes could be substrate specific. Additional experiments would be required in order to confirm this suggestion and to the identity genes that are specifically expressed in response to a given substrate.

It is estimated that a typical mammalian cell expresses about 10,000 to 20,000 mRNA species and in diseased conditions between 0.2–10% of this may be differentially expressed. Considering that approximately half of the human genome is represented on the HG-U133A GeneChip^® ^, the detection of 6,438 genes falls within the expected range. It is also estimated that approximately 10–20% of the entire genome is expressed in any cell type. Our study gave a slightly higher value, with 33.8% of the genes expressed in the ARPE-19 cells. This might reflect the fact that we examined only genes that encode for proteins whose identity and function are known. This group includes many of the common housekeeping genes that are expected to be expressed in most cells, and thus might have a higher probability for detection in our analysis. The group of unidentified genes not included in our analysis may more likely include rare genes that would not be expected to be as widely expressed.

The Functional Gene Expression Profile is not a strict quantitative indicator of any given function because, firstly, it takes into account only the expression of a gene and not its level of expression. Secondly, individual genes might have excitatory or inhibitory influence on a particular function. Lastly, the level of gene expression may not be quantitatively related to the function. Other parameters such as rate of translation, RNA turnover, post-transitional modification and degradation rate of proteins can all affect the degree to which a given gene and its protein product contribute to the functional state of cells. Nevertheless, it is reasonable to assume that if a cell is actively involved in a given function it will likely express many of the genes involved in the corresponding functional pathways. Likewise, if the cell is not involved in a specific function it will express fewer of the genes related to those functionally related pathways. Even though some genes activate and others inhibit the function, a cell must maintain homeostasis and thus is likely to regulate any ongoing activity – for example cell division – by balancing the expression of excitatory and inhibitory factors. If this is the case, all genes within appropriate cell division pathways would have a greater probability of being expressed in an actively dividing cell than in one that is quiescent. The actively dividing cell would have a higher level of "gene chatter" within cell division pathways, which should be reflected in a shift in the percentage of genes expressed within that functional category. An additional aim in developing a Functional Gene Expression Profile was to facilitate analysis of a large microarray data set. The major contribution that our work provides in this regard is the development of a classification scheme of limited size, which includes virtually all ARPE-19 expressed genes whose functions are known. The classification scheme was constructed using Affymetrix search terms (i.e., key words) that appear in the HG-U133A GeneChip^® ^annotations, which provide a readily accessible, standard vocabulary for the uniform classification of gene expression data sets by other investigators. These GeneChip^® ^annotations are updated quarterly by Affymetrix. They can be easily downloaded and used to create or update GeneChip^® ^libraries and searchable relational databases.

The Profile is essentially an expanded pie chart, that contains more information than can feasibly be presented in a standard pie chart format. Nevertheless, it can be displayed in a reasonably sized bar graph with 130 data points. By representing expression results as a percentage of the total number of expressed genes, direct comparisons of expression information (albeit in compressed form) can be made for virtually all functionally identified genes across cell types, treatments, physiological states, etc. Recently, somewhat similar approaches have been used to make data mining SoftWear tools (EASE) that allow comparisons of gene lists and search for gene categories over represented in a sample.

If the Function Gene Expression Profile is to be a useful as a genetic blueprint for cell types or functional states, it must be sensitive enough to reflect substantive differences in gene expression that are unique for those specific cell types or physiological sates. Experiments are underway to prepare Profiles of appropriate data sets from other cell types and to carry out comparative analyses. From these comparisons, we will determine the degree to which Profiles differ, and more importantly, if these differences can provide the basis for identification of genes and functional pathways that are of particular relevance to the cell or physiological state in question. Our current results do show a significant difference in the profile of genes expressed by ARPE-19 compared to the profile of the genes represented on the HG-U133A GeneChip^® ^. Thus, we have one comparison that shows unique aspects of ARPE-19 gene expression compared to all genes expressed by all cells. Even in the absence of further comparative data, the Profile provides a useful gene expression snapshot of a confluent monolayer of ARPE-19 cells. The highest percentages of genes expressed were in categories that could be related to specialized RPE functions (receptors and binding proteins) and those that may be related to housekeeping genes (energy metabolism, transport, and gene/protein expression). The quiescent state of the culture is consistent with low percentages of genes expressed in functional categories that include cell division, cell growth, and cell structure/mobility.

## Conclusion

We present a system of profiling the expressed genes based on their functions. The Profile can be compared with equivalent microarray data from other appropriate samples in order to highlight cell-specific attributes or treatment-induced changes in gene expression. The usefulness of these analyses is based on the assumption that the numbers of genes expressed within a functional category provide an indicator of the overall level of activity within that particular functional pathway.

## Competing interests

The author(s) declare that they have no competing interests

## Pre-publication history

The pre-publication history for this paper can be accessed here:



## Figures and Tables

**Table 1:** Functional Categories that Contain the Largest Percentages of Genes

Categories with ≥ 4% of genes represented on the chip		Categories with ≥ 0.5% of genes expressed by ARPE		
Functional Category	Percentage*	Functional Category	Percentage 1	Percentage 2
	
Cell Sur face Receptors	**9.6**	Binding Proteins	**4.2**	**12.3**
Receptors	**9.6**	Cell Surface Receptors	**2.3**	**6.8**
Binding Proteins	**9.6**	Receptors	**2.3**	**6.8**
Kinase	**5.4**	Kinase	**2.0**	**5.9**
Signal transduction	**5.4**	Hydrolase	**1.9**	**5.7**
Transport (Metabolism)	**5.1**	Transport	**1.9**	**5.6**
Transport	**5.1**	Transport (Metabolism)	**1.8**	**5.6**
Transcription Factor	**4.8**	Signal transduction	**1.7**	**4.9**
Channels/Transport Proteins	**4.2**	Metabolism (signal transduction)	**1.6**	**4.9**
Hydrolase	**4.3**	Metabolism	**1.6**	**4.7**

* number of genes in a given category that are represented on the chip/ total number of genes represented on the chip

1 number of genes in a given category that are expressed by ARPE/ total number of genes represented on the chip

2 number of genes in a given functional category that are expressed by ARPE/ total number of genes expressed
